# Supplementary material for: FAST-MaP: Chemical Mapping of RNA Structures Using Primer-less Sequencing
Source: bioRxiv. 2026 Sep 23:2026.09.22.753544. Preprint. [Version 1] doi: 10.64898/2026.09.22.753544 (PMC13622246; doi:10.64898/2026.09.22.753544)
Supplement: Supplementary file 2 [file media-2.zip › FAST-MaP_ROOL-env120_SupplementaryData/raw_sequencing/plasmidsaurus-pilot.zip!/plasmidsaurus-pilot/reports/DMS.html]

cmuts · Reactivity report

# cmuts · Reactivity report

2 conditions · generated on 2026-09-16 14:25 PDT by cmuts v2.0.0

Offline snapshot: the condition chips work; the reference and channel controls need the server.

Condition:

hepes-DMSbicine-DMS

Reactivity from:

SubstitutionsInsertionsDeletionsTerminationsPresets:

DMSSHAPE

Library

1

References

615

Reference length

Primary Reads

9,987

Counted

7

Rejected

6

Unmapped

Supplementary Reads

0

Counted

1,957

Rejected

Reactivity

0.008

Mean reactivity

0.001

Mean error

SNR

3.61

Mean SNR

Library

1

References

615

Reference length

Primary Reads

9,973

Counted

9

Rejected

18

Unmapped

Supplementary Reads

0

Counted

1,953

Rejected

Reactivity

0.011

Mean reactivity

0.001

Mean error

SNR

4.50

Mean SNR

Sequence

CUUGUGUAUGACUGUCUAAUCAACAGUGCAAGGAAUUAGUUGUGCUCUCAAAAGGAGUUAUGUGAAGGAAAGAUUAAAUGGAUACAUUUAAUUAAGUACAAUUACUACAAAAUCUAUAAUGACUGGGUAAGUCACCCGUAAGAAGGAUGAGUUAGUAGAGAUAUAAUAAUAUCUUACAGUUCAACUGAUGUGCAAGUUUAUAAGUAGACGUAGUGUGAAAGACUUAUCGCUAACGCAAUAGACGUUAUCUCGAAAGGAUAAAAGAAACCAAGAAGAUAUACAAAUUGGUAACUUGUAUAAGCCCUUGGUAAUACCAAAUAAGUUAGUUCCUCAUGAUGUCGUGGAAACAUGACUAUAAGAUAAGUUGAAUUCGAUAGUAGCCCAAGAGGAAUCAAAUGAGUAAAACAAGUAUUAUAGGUAAAUAUUAUGUUUUUCUAUCUGAAUGAUUGUGCCUUGAAAAAGGUGGGUGAGGUCUGUGGGUAAAAAUUAAUUCCCAUCUGGUUUUGGAUUUUCUUCGGAAAAUUGGUGCAAGAAGCGUGGAGUUGCUAACCAUUGCUCAGACUUGUAUCGCCAGUAGCACGAUAACGAUAUUUAUGAAUGUUGUAAGGUGAAUAA

Reactivity profile

010020030040050060000.050.10.150.2

ReactivityStd. errorResidueReactivity

Coverage

010020030040050060002k4k6k8k10k

ResidueCoverage

Read lengths

200400600800100012000500100015002000

Read lengthReads

Pairwise correlation

Pairwise conditional

Pairwise coverage

Multi-plot

hepes-DMSbicine-DMS−

+ Add profile

010020030040050060000.050.10.150.2

hepes-DMSResidueReactivity

Difference plot

hepes-DMSbicine-DMS

minus

hepes-DMSbicine-DMS

0100200300400500600−0.01−0.00500.0050.01

ResidueReactivity Difference
